# Acute Coronary Syndrome in Intensive Care Unit Patients: Troponin or Triglyceride Glucose Index Levels?

**DOI:** 10.3390/jcm14207421

**Published:** 2025-10-21

**Authors:** Özgen Şafak, Mehmet Tolga Hekim, Didar Elif Akgün

**Affiliations:** Department of Cardiology, Balikesir University Faculty of Medicine, 10185 Balikesir, Türkiye; ozgen_safak@yahoo.com (Ö.Ş.);

**Keywords:** TyG index, triglyceride-glucose index (TyG), insulin resistance, cardiovascular risk, cardiovascular disease, acute coronary syndrome, troponin, myocardial infarction

## Abstract

**Background and Objectives:** There are few studies suggesting that the Triglyceride–Glucose Index (TyG), which is mostly defined as a predictor of diabetes, can be used as a predictor of coronary artery disease. In this study, we investigated the relationship between TyG index and acute coronary syndrome (ACS). **Materials and Methods:** Patients who were hospitalized in the coronary intensive care unit between January 2023–December 2024 were included in the study regardless of the admission diagnosis. ACS defined as ST elevation myocardial infarction (STEMI) and non-STEMI. The TyG index was calculated with the formula LN [fasting triglycerides (mg/dL) × fasting glucose (mg/dL)/2]. The relationship between the presence of acute coronary syndrome and troponin level was compared with the TyG index. **Results:** A total of 586 individuals, 353 (60.2%) males and 233 (39.8%) females, were included in this study. The mean TyG index value was calculated as 75 ± 0.31 (4.03–5.99). ACS was detected in 36.9% (n = 216) of the participants. The mean TyG index was higher in the group with ACS (4.92 ± 0.29) than in the group without ACS (4.65 ± 0.27), *p* < 0.001). Similarly, the mean value of triglyceride (171.58 ± 114.45 vs. 120.92 ± 63.02, CI 95%, *p* < 0.001) and glucose (133.57 ± 48.87 vs. 104.88 ± 34.76, CI 95%, *p* < 0.001) were also higher in the group with acute coronary syndrome. In logistic regression analysis, the TyG index was identified as the most significant predictor of ACS, associated with a 30.994-fold increase in ACS probability. **Conclusions:** This study demonstrated that the TyG index is a significant predictor of acute coronary syndrome independent of the hospitalization reason. The TyG index can be used as a valuable marker in clinical practice because it includes modifiable risk factors for coronary artery disease.

## 1. Introduction

Coronary artery disease is one of the leading causes of mortality, particularly in developed countries, largely attributable to the high prevalence of metabolic syndrome. Key elements of metabolic syndrome—such as abdominal obesity, elevated systolic blood pressure, impaired fasting glucose, high triglyceride levels, and low high-density lipoprotein (HDL) cholesterol—constitute major risk factors for cardiovascular disease [1]. Insulin resistance is commonly linked to hyperglycemia, dyslipidemia, and hypertension, and plays a pivotal role in the early stages of atherosclerosis through its procoagulant and inflammatory effects [2]. Consequently, the timely identification and management of insulin resistance and dyslipidemia are critical for the prevention of coronary artery disease.

The triglyceride–glucose (TyG) index is a simple, cost-effective, reliable, and reproducible marker of insulin resistance, which was first proposed in 2008 [3]. Previous studies have demonstrated that the TyG index may serve as a predictor of Type 2 diabetes mellitus (DM) development in non-diabetic individuals [4]. Furthermore, in patients with established Type 2 DM, the TyG index shows a positive correlation with HbA1c levels and the Homeostasis Model Assessment of Insulin Resistance (HOMA-IR), exhibiting a linear dose–response relationship with HOMA-IR risk, thereby supporting its role as a follow-up indicator [5].

A growing body of evidence suggests that the TyG index may also serve as a marker for cardiovascular risk. It has been demonstrated the TyG index possess superior predictive power for identifying stable coronary artery disease compared to isolated triglyceride or glucose measurements [6]. A meta-analysis further corroborated the TyG index as an independent predictor of cardiovascular events, regardless of diabetic status [7]. A large retrospective observational cohort study of 5,593,134 individuals found that a higher TyG index was associated with increased risks of diabetes mellitus, coronary artery disease, cardiovascular diseases, atrial fibrillation, diabetic complications, acute pancreatitis, myocardial infarction (MI), stroke, and COVID-19 complications, independent of conventional risk factors such as age, sex, smoking status, alcohol consumption, physical activity, total cholesterol, body mass index (BMI), and systolic blood pressure [8]. Additionally, a significant association between multivessel coronary artery disease and the TyG index has been reported [9].

The impetus for the present study originated from clinical observations at our institution. We noted significant coronary lesions and extensive plaque burden, discordant with traditional risk factor profiles, in a subset of patients under 60 years of age presenting with ACS. This prompted a preliminary prospective screening of 52 patients over one month, which indicated that the TyG index tended to approach a value of 5.0 in ACS patients. Building upon this initial finding, we conducted a larger retrospective analysis of a two-year cohort to systematically investigate the correlation between the TyG index and troponin levels in diagnosing ACS. This study aims to evaluate the predictive power of the TyG index for ACS in a broad population of patients admitted to the coronary and emergency intensive care unit (ICU).

## 2. Methods

### 2.1. Study Design and Population

This cross-sectional descriptive study enrolled patients admitted to the coronary ICU and emergency ICU of Balikesir University between January 2023 and December 2024, irrespective of diagnostic differences. The patient data were retrospectively obtained from hospital records. The association between the TyG index, ACS, and troponin levels was the primary focus. ACS was defined as either STEMI or NSTEMI. Patients diagnosed with unstable angina pectoris were excluded due to potential unreliability in clinical history and documentation.

Exclusion criteria encompassed isolated decompensated heart failure, severe renal or hepatic dysfunction, acute renal failure, pregnancy, congenital heart disease, active endocarditis, rheumatic diseases, active non-ischemic bleeding, ongoing infections, sepsis, malignancy, acute cerebrovascular events, angiographically normal coronary arteries, a body mass index (BMI) > 45 kg/m^2^, and suspected familial hypertriglyceridemia (triglycerides > 500 mg/dL). After evaluating the records of 864 patients, 586 individuals with complete datasets were included in the final analysis.

### 2.2. Data Collection and Definitions

Blood samples obtained during hospitalization were utilized for the study. Biochemical evaluations except for troponin were conducted using peripheral blood samples collected at 7 a.m. following at least an 8 h fasting period at the end of the patient’s one night hospitalization. As patients diagnosed with acute renal failure were not included in this study, the first creatinine values of hospitalized patients were analyzed.

The highest troponin level recorded within the first 48 h of hospitalization was used for analysis. Cases with positive troponin levels were classified as ACS. A single troponin test was used in all patients included in the study, and all blood samples were processed using a standardized method. The troponin reference ranges were the same for all patients.

The TyG index was calculated using the following formula: LN [fasting triglycerides (mg/dL) × fasting glucose (mg/dL)/2]. A TyG index value of 4.49, corresponding to the established cutoff for insulin resistance, was used to dichotomize patients into low and high TyG groups.

Additional data extracted from patient records included: presence of coronary artery disease, peripheral artery disease, diabetes mellitus (defined as HbA1C ≥ 6.5% or use of anti-diabetic medications), hypertension (blood pressure > 140/90 mmHg or use of antihypertensive drugs), prior cerebrovascular events, smoking status, alcohol use, and medication history. BMI was calculated using the formula: weight (kg)/height (m^2^).

The study received approval from the Local Ethics Committee. No artificial intelligence (AI)-assisted technologies (e.g., large language models, chatbots, or image generators) were employed at any stage of this research.

### 2.3. Statistical Analysis

Statistical analyses were conducted using IBM SPSS Statistics v26.0 (IBM Corp., Armonk, NY, USA). The normality of variable distributions was assessed through both visual methods (histograms) and analytical tests (Kolmogorov–Smirnov and Shapiro–Wilk). Descriptive statistics were presented as frequencies and percentages, as well as means with standard deviations or minimum–maximum ranges. Depending on the nature of the data, the Chi-square test, independent *t*-test, or Mann–Whitney U test were employed. Correlation analyses between variables were performed using either the Pearson or Spearman correlation coefficients, based on distribution characteristics. The relationship between the TyG index and ACS was evaluated using logistic regression modeling. The predictive performance of the TyG index for ACS was analyzed via receiver operating characteristic (ROC) curves, and the area under the curve (AUC) was calculated. A type I error level of α = 0.05 was considered statistically significant.

## 3. Results

A total of 586 individuals were included in the study, comprising 60.2% (n = 353) males and 39.8% (n = 233) females. The mean age of all participants was 67.37 ± 11.55 years (range: 24–92 years). Table 1 summarizes the medical history and medications used by the patients. 50.7% (n = 297) of the patients had diabetes mellitus, and 60.2% (n = 353) had hypertension.

The median troponin level for the entire population was 28.35 ng/mL (range: 2.30–27 ng/mL) (Table 2), with median values of 18.90 ng/mL (2.3–25.6 ng/mL) in females and 36 ng/mL (2.3–27 ng/mL) in males. The median troponin was significantly higher in the ACS group (424.80 ng/mL) compared to the non-ACS group (13.85 ng/mL). Descriptive statistics for troponin are detailed in Table 2.

The mean fasting triglyceride and glucose levels were 139.60 ± 88.98 and 115.45 ± 42.80 mg/dL, respectively. The mean TyG index was 4.75 ± 0.31 ACS was diagnosed in 36.9% (n = 216) of participants. Coronary angiography had been performed in 55.6% (n = 326) of the participants within six months of hospital admission.

The association between the presence of ACS and TyG index, glucose, and triglyceride levels is summarized in Table 3. The mean TyG index was significantly higher in the ACS group (4.92 ± 0.29) compared to the non-ACS group (4.65 ± 0.27; 95% CI: 0.22–0.32; *p* < 0.001). Likewise, both triglyceride (171.58 ± 114.45 vs. 120.92 ± 63.02 mg/dL; 95% CI; *p* < 0.001) and glucose (133.57 ± 48.87 vs. 104.88 ± 34.76 mg/dL; 95% CI; *p* < 0.001) levels were significantly elevated in patients with ACS.

The prevalence of ACS was significantly higher in the group with a TyG index > 4.49. A significantly greater proportion of individuals with a high TyG index exhibited elevated troponin. Moreover, the mean TyG index was significantly higher among participants with troponin > 35 ng/mL (4.87 ± 0.30 vs. 4.65 ± 0.27; 95% CI: 0.17–0.26; *p* < 0.001) (Table 4).

Correlation analysis conducted among patients with ACS and troponin > 35 ng/mL revealed a positive correlation between glucose, triglyceride and TyG index. Among these variables, the TyG index showed a stronger correlation with troponin level than either glucose or triglyceride levels alone (Table 5).

Logistic regression analysis identified the TyG index as the most significant predictor of ACS. While glucose and triglyceride levels were associated with 1.001-fold and 0.998-fold increases in risk, respectively, the TyG index demonstrated a markedly higher association—corresponding to a 30.994-fold increase in the likelihood of ACS (Table 6).

**Figure 1:**
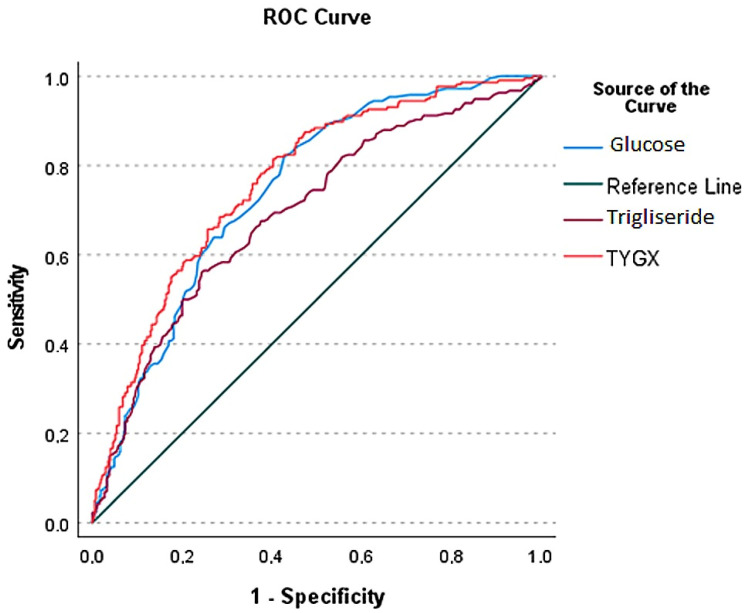
ROC-analysis demonstrating the diagnostic significance of TyG index, glucose and triglyceride in the entire population.

ROC analysis was conducted to compare the predictive abilities of fasting blood glucose, triglycerides, and the TyG index for ACS. The TyG index demonstrated the highest discriminatory power, with an AUC of 0.764 (95% CI: 0.725–0.803, *p* < 0.001). The optimal cut-off value for the TyG index was determined to be 4.75, yielding a sensitivity of 79% and a specificity of 70% (Figure 1).

## 4. Discussion

The triglyceride-glucose index is a robust and independent indicator of ACS, regardless of the reason for admission, demonstrating a statistically significant, moderate correlation with troponin levels. Troponin is a proven marker for the diagnosis and follow-up of acute coronary syndrome. Its place in clinical use is clear. However, it has no predictive value for the development of acute coronary syndrome in asymptomatic coronary artery disease. The TyG index is superior to troponin level as a predictor of acute coronary syndrome in chronic coronary artery disease. Specifically, at values exceeding 5.0, the TyG index may offer enhanced risk stratification over troponin alone in NSTEMI patients with only mild troponin elevation. In an era of growing emphasis on preventive cardiology, these findings provide valuable insights for clinical decision-making.

The prevention of metabolic syndrome is a cornerstone of reducing coronary artery disease risk, with diabetes and dyslipidemia representing its most significant modifiable components. The link between insulin resistance and coronary artery disease is well-documented [10]. Although HOMA-IR is a widely used measure of insulin resistance, it requires additional testing and incurs higher costs, limiting its routine clinical application. HOMA-IR has been shown to correlate with coronary artery disease in both obese and non-diabetic individuals [11]. In addition, metabolic score for insulin resistance (METS-IR) and quantitative insulin sensitivity check index (QUICKI) are markers whose relationship with metabolic syndrome has been investigated. QUICKI has been shown to be associated with metabolic syndrome and can be used as a metabolic syndrome marker [12]. Additionally, it has been shown that METS-IR, which is associated with intravisceral, intrahepatic and intrapancreatic fat levels, can be used as a cardiovascular risk indicator [13,14]. In contrast, the TyG index offers a fast, cost-effective, and reliable alternative that is more accessible for widespread clinical use [3]. Particularly in the early stages, the TyG index is recommended as a practical substitute for HOMA-IR in predicting diabetes. Furthermore, it may be used for monitoring treatment adherence over time. Supporting the findings of this study, Da Silva et al. reported a significant association between symptomatic coronary artery disease and elevated TyG index values [15]. Additionally, the TyG index was positively correlated with several cardiometabolic risk factors, including BMI, waist circumference (WC), total cholesterol, low-density lipoprotein (LDL) cholesterol, and elevated systolic blood pressure. There is also a positive correlation between dyslipidemia, diabetes, hypertension, physical inactivity, and smoking with TyG index. The highest TyG values were observed in individuals whose carbohydrate intake exceeded 65% of total daily energy. Consequently, the study recommended reducing carbohydrate consumption as a preventive measure [15].

Previous studies demonstrate the strong correlation between coronary artery calcium (CAC) score-coronary artery disease severity and TyG index [16]. Research also indicated that the TyG index is an independent predictor of CAC progression and provides incremental risk stratification in low-risk populations [17]. In these studies, calcified and mixed plaque occurrence was associated with increased TyG index, and the authors correlated the occurrence of increased acute coronary syndrome events and major adverse cardiovascular events (MACE) with this theorem [18]. Li Pan et al. worked on the relationship and prognostic value of the TyG index in patients with acute coronary syndrome by using optical coherence tomography [19]. They obtained the optical flow ratio (OFR) of the patients and, by using the TyG index, divided them into three groups as T1-T2-T3 (<7.42, 7.42–7.95, and >7.95 retrospectively). Although they conducted a study with high TyG results by performing the division operation in the TyG formula in the wrong order, changes were observed in OCT results in parallel with the increase in TyG. They found that as the TyG index increased, the proximal location of the culprit lesion and major adverse cardiovascular events increased, and the optical flow ratio decreased. These findings were statistically significant between three groups. As expected in group T3 has the highest TyG index value and the lowest post-procedural OCT results [19]. In studies evaluating the relationship between the TyG index and long-term mortality, it was found that the group with a high TyG index was associated with more MACE and lower event-free survival [6,20]. Although the reason is not fully understood, decreased coronary collateral flow and increased chronic total occlusion were detected in the group with high TyG index [21]. Decreased coronary collateralization in patients with metabolic syndrome may be associated with decreased proangiogenic growth factors and production of reactive oxidative species (ROS). Furthermore, ongoing endothelial dysfunction is also thought to contribute to this pathology [22,23].

Marik and Bellomo provide a theoretical framework of this situation as an underlying pathophysiology and named it the ‘stress hyperglycemia survival hypothesis’. Stress-induced hyperglycemia is defined as blood glucose levels exceeding 180 mg/dL during acute stress in patients without a previous diagnosis of diabetes [24]. Sympathoadrenal axis activation during the acute phase maintains energy supply to immune and neuronal cells through hepatic gluconeogenesis and insulin resistance [25]. Glucose homeostasis is impaired by the influence of catecholamines, cortisol, glucagon and growth hormone [26]. To enhance glucose transport, upregulation of Glucose Transporter Type 1 expression is the another mechanism of this process, which leads to regulation of immune function by blood glucose levels and causes bidirectional immunosuppression [26,27]. Blood glucose regulation is severely impaired as a result of glucose transporter type 4 (GLUT-4)-mediated glucose uptake into skeletal muscle, deterioration in hepatic gluconeogenesis and an increase in inflammatory cytokines [26]. At this stage, studies have been conducted on the effects of stress-related hyperglycemia and diabetic hyperglycemia on mortality in acute illnesses. In trauma patients, stress-related hyperglycemia has been found to be associated with higher mortality than diabetic hyperglycemia [28]. The effect of hypoglycemia on mortality should also be taken into account in stress-related hyperglycemia [25]. In anticipation of this eventuality, efforts were undertaken to obtain data from periods outside of patient hospitalization for acute coronary syndrome. Glucose levels were shown to rise in patients with ST-segment elevation myocardial infarction, although triglyceride levels experienced a modest decline. Research about this situation was considered appropriate to plan. Nevertheless, it is important to acknowledge that the cohort of STEMI patients [29] in the study is very limited. The median and mean troponin levels (28.35 ng/mL, 1460 ng/mL) indicate a considerable group of patients lacking severe myocardial damage.

A study evaluating the long-term follow-up of STEMI and non-STEMI patients, which included 23,270 patients, showed that 1-year outcomes were similar in both groups. Cardiovascular risk factors, excluding diabetes, and the rate of repeat revascularization were found to be higher in non-STEMI patients [30]. Contrary to the low early mortality in non-STEMI patients, the increase in long-term mortality at one-year follow-up has been linked to the difficulty of long-term recovery in non-STEMI patients due to multiple comorbidities. In addition, failure to provide optimal coronary artery disease treatment, especially antiplatelet therapy, due to multiple comorbidities and multiple drug use in non-STEMI patients has been associated with long-term mortality being similar or worse than in STEMI patients [31,32,33]. Among patients with ACS, the TyG index has been assessed in relation to MACE [34], the prevalence of coronary artery disease [35], post-procedural cardiac function [36], and clinical prognosis [37]. The present study contributes uniquely by demonstrating that the TyG index can predict ACS across a broader hospitalized population, irrespective of admission reason. The TyG index was found to predict ACS nearly 30 times more strongly than elevated glucose or triglyceride levels alone. These findings underscore the utility of the TyG index as a powerful and practical clinical tool for ACS risk stratification and therapeutic planning. The widespread use of high-sensitivity cardiac troponin (hs-cTn) allows for the clinical detection of mild myocardial damage, and the increased clinical use of angiography may be associated with increased angiography-related complications and mortality [38]. Therefore, studies are currently underway to exclude critical coronary artery disease using coronary CT angiography instead of diagnostic coronary angiography in patients without significant elevations in hs-cTn [39]. Considering the significantly increased risk of contrast-induced nephropathy in non-STEMI patients with multiple comorbidities, it is clear that widespread use of coronary CT angiography would not be beneficial in these patients. TyG index is a marker that can be used in this patient group.

The fact that cardiovascular risk factors other than diabetes were detected more significantly in non-STEMI patients [23] also explains the superiority of the TyG index over glucose and triglyceride levels in non-STEMI patients. In this regard, the current study distinguishes itself from previous investigations. Nevertheless, the optimal cut-off value for the TyG index in clinical practice remains controversial. Various studies have proposed differing thresholds, yet no universal consensus has emerged to date [28,33,34].

This study is limited by the fact that not all patients received coronary angiography during their hospital admission. However, all patients diagnosed with ACS had undergone angiography within the preceding six months. Given the study’s hypothesis and objectives, immediate angiography was not deemed essential. Another limitation is the sample size; conducting the study in a larger cohort would enhance the clinical validity and generalizability of the findings.

## 5. Conclusions

A TyG index exceeding 4.75, which may arise from mildly elevated glucose and triglyceride levels that often go unnoticed, can be considered a significant risk marker for acute coronary syndrome (ACS), regardless of the hospitalization cause. Additional large-scale and targeted studies are needed to identify the most accurate cut-off value for the TyG index in predicting ACS. The TyG index has the potential to be a valuable tool in clinical practice, as it seems to be more predictive than traditional risk markers like glucose or triglyceride levels alone. It is anticipated that its use may be beneficial for the diagnosis and risk assessment of NSTEMI patients, especially those with mild troponin elevation.

## Figures and Tables

**Table 1:** Medical History and Medications of the Patients.

	ACS (+) 36.9% (n = 216)	ACS (−) 63.1% (n = 370)	All Patients (n = 586)	*p*
Age (mean ± SD)	65.13 ± 11.54	68.67 ± 11.38	67.37 ± 11.55	*p* < 0.001
Female Gender	73 (33.8%)	160 (43.2%)	233 (39.8%)	*p* < 0.001
HT	128 (59.3%)	225 (60.8%)	353 (60.2%)	*p* > 0.05
DM	105 (48.6%)	192 (51.9%)	297 (50.7%)	*p* > 0.05
HF	59 (27.4%)	135 (36.5%)	194 (33.2%)	*p* > 0.05
Stroke	3 (1.4%)	14 (3.8%)	17 (2.9%)	*p* > 0.05
COPD	12 (5.6%)	47 (12.7%)	59 (10.1%)	*p* > 0.05
CKD	20 (9.3%)	48 (13.0%)	68 (11.6%)	*p* > 0.05
AF	27 (12.6%)	144 (39.1%)	171 (29.4%)	*p* < 0.001
Bleeding	4 (1.9%)	7 (1.9%)	11 (1.9%)	*p* > 0.05
Antiaggregant	168 (77.8%)	173 (46.9%)	341 (58.3%)	*p* < 0.001
Anticoagulant	45 (20.8%)	166 (45%)	211 (36.1%)	*p* < 0.001
Statin	118 (54.6%)	125 (33.8%)	243 (41.5%)	*p* < 0.001
OAD	49 (22.7%)	87 (23.5%)	136 (23.2%)	*p* > 0.05

AF: Atrial Fibrillation, CKD: Chronic Kidney Disease, COPD: Chronic Obstructive Pulmonary Disease, DM: Diabetes Mellitus, HF: Heart Failure, HT: Hypertension, OAD: Oral Antidiabetic Drug. Note: Data are expressed as n (%) excluding age.

**Table 2:** Troponin-I descriptives.

	N	Q1 (25%)	Median	Q3 (75%)	IQR
**Troponin ACS+**	216	106.1	424.80	3268	3162
**Troponin ACS−**	370	7.6	13.85	26.25	18.65

**Table 3:** Mean Glucose, triglyceride, and TyG levels in patients with or without acute coronary syndrome.

	ACS (+)	ACS (−)	*p*
**TyG index**	4.92 ± 0.29	4.65 ± 0.27	*p* < 0.001
**Triglyceride (mg/dL)**	171.58 ± 114.45	120.92 ± 63.02	*p* < 0.001
**Glucose (mg/dL)**	133.57 ± 48.87	104.88 ± 34.76	*p* < 0.001

Note: Data are presented as mean ± standard deviation.

**Table 4:** TyG index of Whole Acute Coronary Syndrome Group and Troponin > 35 ng/mL Group.

	TyG Index ≤ 4.49 n = 131 (22.4%)	TyG Index > 4.49 77.6% (n = 455)	*p*
**ACS Patient**	n = 13 (9.9%)	n = 203 (44.6%)	*p* < 0.001
**Troponin** **>35 ng/mL**	n = 24 (18.3%)	n = 239 (52.5%)	*p* < 0.001

Note: Data are expressed as n (%).

**Table 5:** Correlation analysis of participants with ACS and Troponin >35 ng/mL.

	r	*p*
**TyG index**	0.430	<0.001
**Glucose**	0.397	<0.001
**Triglyceride**	0.321	<0.001

**Table 6:** Logistic regression analysis in patients with acute coronary syndrome.

Variable	RR	95% CI	*p*
**Age**	0.977	0.958–0.997	0.025
**Gender**	0.819	0.511–1.313	0.407
**Troponin**	1.002	1.002–1.003	<0.001
**Triglyceride**	0.998	0.993–1.003	0.338
**Glucose**	1.001	0.992–1.009	0.869
**TyG index**	30.994	4.217–227.775	<0.001

## Data Availability

The data supporting the findings of this study are available from the corresponding author upon reasonable request.

## Abbreviations

ACS	Acute Coronary Syndrome
AI	Artificial Intelligence
AUC	Area Under the Curve
BMI	Body Mass Index
CAC	Coronary Artery Calcium
DM	Diabetes Mellitus
HDL	High-Density Lipoprotein
HOMA-IR	Homeostasis Model Assessment of Insulin Resistance
ICU	Intensive Care Unit
MACE	Major Adverse Cardiovascular Events
METS-IR	Metabolic Score for Insulin Resistance
QUICKI	Quantitative Insulin Sensitivity Check Index
ROC	Receiver Operating Characteristic
STEMI	ST Elevation Myocardial Infarction
TyG	Triglyceride–Glucose
WC	Waist Circumference
